# The ABCs of Stargardt disease: the latest advances in precision medicine

**DOI:** 10.1186/s13578-024-01272-y

**Published:** 2024-07-26

**Authors:** Yasmine A. Zaydon, Stephen H. Tsang

**Affiliations:** 1 Departments of Ophthalmology, Pathology and Cell Biology, Jonas Children’s Vision Care, and Bernard and Shirlee Brown Glaucoma Laboratory, Columbia Stem Cell Initiative, Institute of Human Nutrition, Vagelos College of Physicians and Surgeons, Columbia University, New York, NY USA; 2 Edward S. Harkness Eye Institute, New York-Presbyterian Hospital, New York, NY USA; 3 Department of Pathology and Cell Biology, The Herbert Irving Comprehensive Cancer Center, Institute of Human Nutrition, Columbia University, New York, NY USA

**Keywords:** ABCA4, Stargardt disease, Gene therapy, Drug therapy, Stem cell therapy

## Abstract

Stargardt disease (STGD) is the most common form of inherited juvenile macular dystrophy and is caused by sequence variants in the *ABCA4* gene. Due to its genetic complexity and phenotypic variability, STGD poses significant therapeutic challenges. In the past decade, a lot of progress has been made regarding our understanding of the molecular and clinical aspects of STGD, along with its mechanisms. This has led to the development of new therapies, and there are human clinical trials currently ongoing. This paper evaluates the emergence of pharmacological approaches targeting the visual cycle to mitigate retinal damage, the role of gene therapy in correcting specific genetic defects, and the use of stem cell therapies aimed at retinal regeneration by showcasing the latest clinical trials and precision medicine approaches.

## Introduction

STGD presents a clinical reality where patients experience a gradual decrease in vision, beginning from the central field and extending outwards. This inherited condition is characterized by progressive central vision loss due to the accumulation of toxic material in the retina. It was named after the German pioneering ophthalmologist Karl Stargardt, who described seven patients with an inherited macular dystrophy in 1909. Macular dystrophy refers to a group of inherited eye disorders that primarily affect the macula, the central part of the retina responsible for sharp, detailed, central vision. This inherited retinal disease (IRD) has a prevalence of ~ 1:1380 individuals, with about 35,000 people affected in the U.S. and 5.5 million affected worldwide [1]. IRDs are a group of genetic disorders that result in progressive loss of vision and can eventually lead to blindness. They include conditions such as retinitis pigmentosa (RP), Leber’s congenital amaurosis (LCA), cone-rod dystrophy, and Stargardt disease, among others [2]. STGD alone accounts for 12% of IRD-related blindness registrations, which refer to the official records of individuals who have been legally registered as blind or visually impaired due to IRDs [3]. However, it was not until more recent years, with advancements in genetics and molecular biology, that the specific genetic mutations associated with STGD were identified. This introductory section outlines the fundamental aspects of STGD, starting with a clinical overview of its impact on central vision and tracing its historical identification. It will then transition into a discussion on the genetic underpinnings of STGD, focusing on the pivotal role of the *ABCA4* gene, and conclude by highlighting the current state of therapeutic approaches and the promise they hold for the future. This section aims to establish a foundational understanding of STGD’s pathophysiology and the genetic complexities that inform the direction of emerging treatments.

## Genetic basis of STGD

**Fig. 1:**
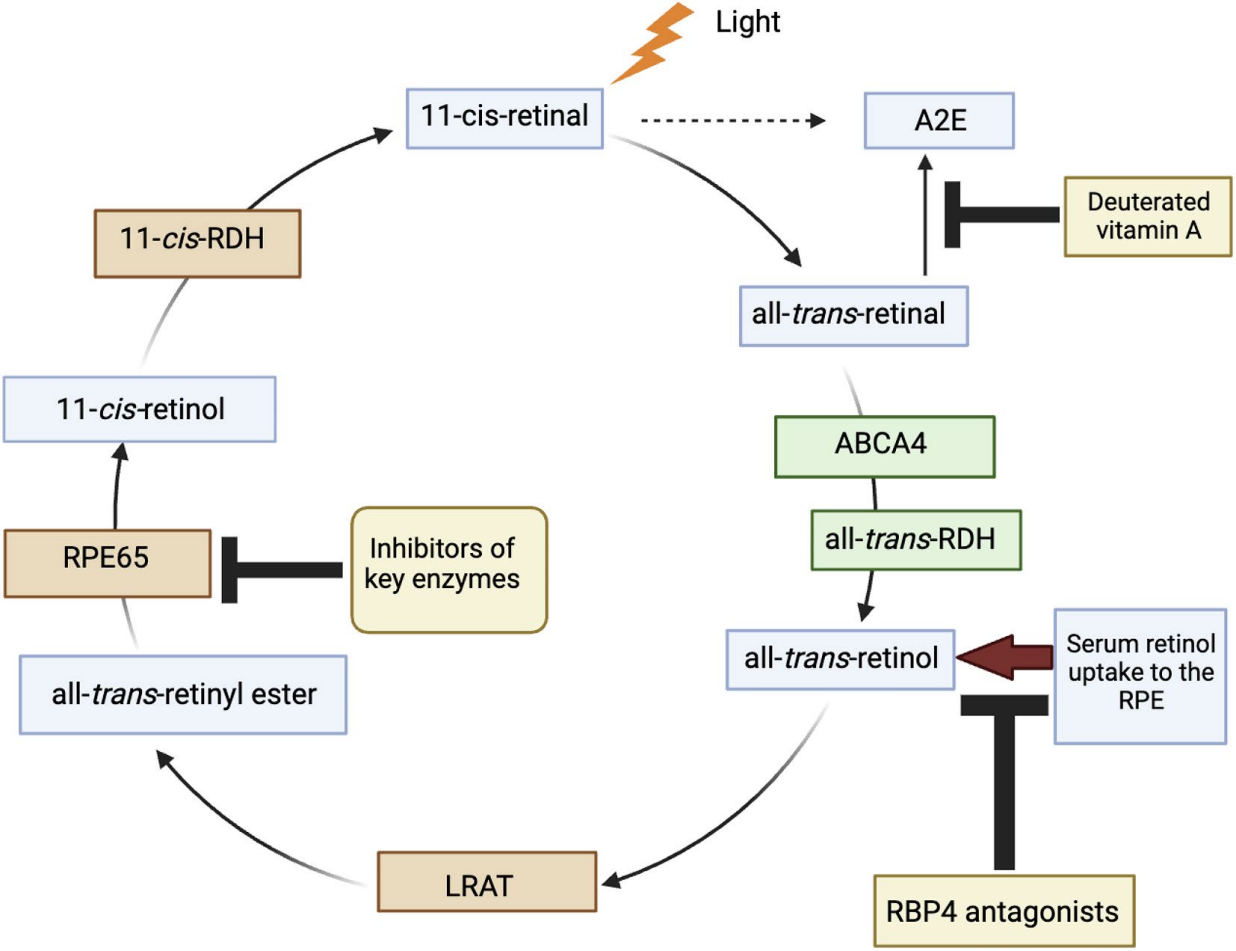
The conversion of light to 11-*cis*-retinal and its subsequent transformation to all-*trans*-retinal involves the *ABCA4* protein. Points of intervention are indicated where inhibitors of key enzymes, deuterated vitamin A, and RBP4 antagonists can reduce the formation of toxic A2E. Abbreviations: *ABCA4*, retina-specific ABC transporter; all-*trans*-*RDH*, all-*trans*-retinol dehydrogenase; *LRAT*, lecithin-retinol acyltransferase; 11-*cis*-RDH, 11-*cis*-retinol-dehydrogenase.

The *ABCA4* gene, located on chromosome 1, was identified as the gene of interest in the inheritance of this disease by a team of researchers led by Rando Allikmets at the National Human Genome Research Institute in 1997 [4]. This gene is named for its product, the ATP-binding cassette, sub-family A, member 4 protein. It is part of the ATP-binding cassette (ABC) transporter superfamily, which is a large family of proteins that utilize the energy derived from ATP hydrolysis to transport various molecules across cellular membranes. The “A4” designation indicates that it is the fourth gene in the “A” sub-family of this group [5]. In healthy eyes, the *ABCA4* gene is crucial because it encodes the ABCA4 protein which plays a major role in the visual cycle, the biochemical cycle of processes enabling vision [6] (Fig. 1). Specifically, *ABCA4* is located in the outer segments of photoreceptor cells, where it actively transports vitamin A derivatives across the disc membranes of the photoreceptor outer segments after phototransduction, the process by which light is converted into electrical signals by the photoreceptors [7]. During phototransduction, one of the byproducts formed is N-retinylidene-phosphatidylethanolamine (N-retinylidene-PE). In a functioning retina, the ABCA4 protein helps to clear this byproduct, preventing its accumulation. However, in individuals with mutations in the *ABCA4* gene, the protein’s function is compromised, leading to an accumulation of toxic retinal substances such as lipofuscin, which is particularly rich in a component called A2E [8]. Lipofuscin is a complex mixture of lipid-containing residues that accumulate as byproducts of cellular metabolism. In the retina, lipofuscin accumulates abnormally in the retinal pigment epithelium (RPE) cells due to the inefficient processing and removal of phototransduction byproducts [9]. This accumulation is toxic to the retinal cells and is the main cause of the progressive vision loss observed in STGD.

### Current and future therapeutic approaches

This review will explore the emerging therapeutic approaches for Stargardt disease, including drug, gene, and stem cell therapies. Each of these therapies addresses different mechanisms of disease and stages of progression. Gene therapy is often most effective before significant cell loss has occurred, aiming to correct or compensate for the underlying genetic defect [10]. Drug therapy can be used both for early intervention and for managing progression in later stages, focusing on preserving remaining retinal function and slowing further degeneration. Stem cell therapy is seen as a possible solution for later stages of IRDs, where the goal is to replace lost cells and restore some visual function. Together, these therapies represent a comprehensive approach to tackling the complex challenge of IRDs, offering hope for preserving and potentially restoring vision in affected individuals. This context is foundational for comprehending the significance of recent scientific progress and ongoing clinical trials.

### Macular degeneration

There are six types of macular dystrophies: STGD, Best disease (BD), X-linked retinoschisis (XLRS), autosomal dominant drusen (ADD), Sorsby fundus dystrophy (SFD) and pattern dystrophy (PD) [11]. Of the six, STGD is the most common and usually occurs during childhood. It may progress rapidly over a few months or gradually over several years. However, in the broader context of all eye diseases, its prevalence is lower than conditions such as age-related macular degeneration (AMD) or cataracts, which are more common in the general population due to their association with aging. In terms of treatment approaches, there is a significant overlap between those being developed for STGD and other inherited conditions leading to macular degeneration. This overlap is primarily because many of these conditions share common pathological mechanisms, such as the death of photoreceptor cells or the dysfunction of the RPE.

While STGD is a focal point in IRD research because of its genetic basis, particularly mutations in the *ABCA4* gene, it is important to recognize the broader context of retinal pathologies. Non-inherited factors such as smoking and prolonged exposure to high-intensity light also contribute significantly to retinal conditions such as AMD [12]. In the spectrum of inherited retinal dysfunctions, STGD is among several, including RP and LCA, each with unique genetic causes and manifestation patterns. The intensity of research and clinical trials varies across different eye diseases, often influenced by prevalence and public health impact [13]. While STGD receives conside=/rable attention due to its prevalence among juvenile macular dystrophies, it may not match the research volume dedicated to conditions such as AMD, which affects a larger segment of the aging population. Nevertheless, STGD research shares methodologies such as gene and stem cell therapy with other eye disease studies, benefiting from and contributing to the wider field of ophthalmic research.

**Fig. 2:**
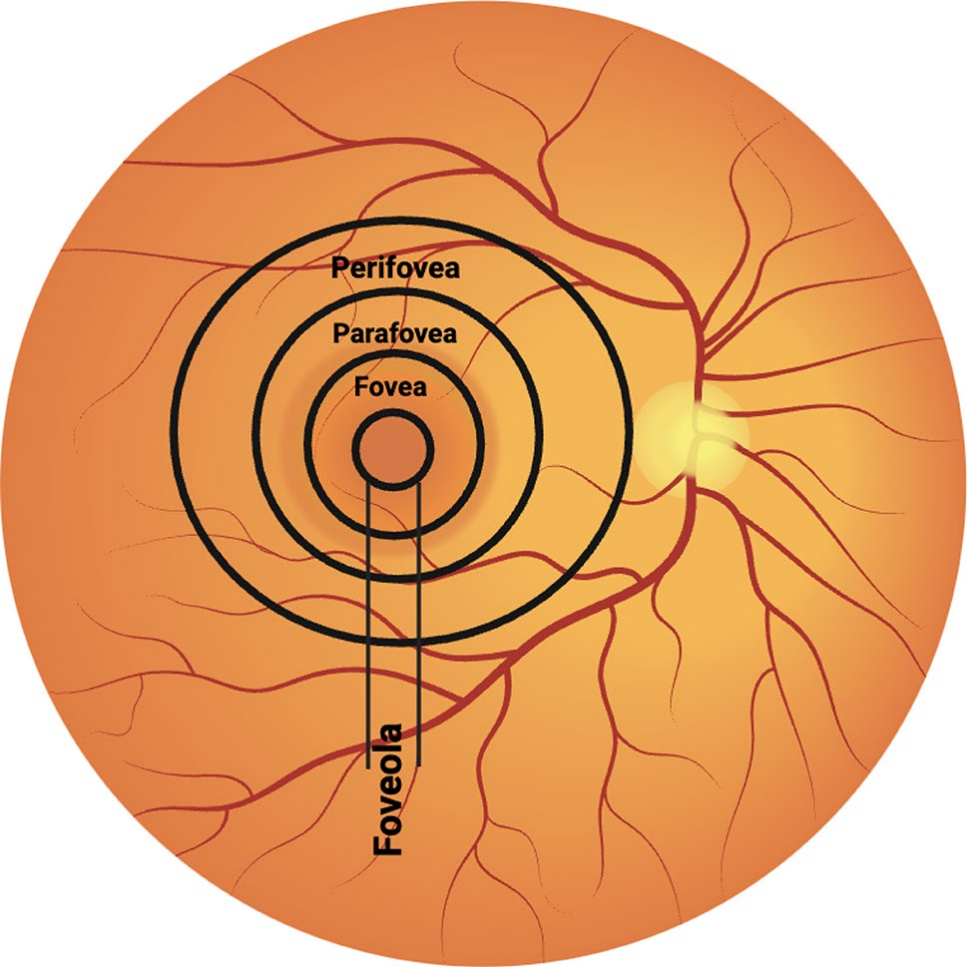
The macula, highlighted here, is made up of the fovea, parafovea, perifovea, and foveola—crucial regions for high-resolution central vision. In STGD, the *ABCA4* gene is mainly expressed in the photoreceptor cells of these regions. Defective ABCA4 protein impairs the clearing of phototransduction byproducts, leading to lipofuscin accumulation, predominantly in the fovea where cell density and visual acuity are highest. This accumulation causes photoreceptor and RPE cell damage, which severely affects central vision and tasks such as reading and driving, while peripheral vision remains less affected.

STGD is characterized by a gradual progression of central vision loss, and visual acuity (VA) varies, ranging from 20/20 to 20/400 [14]. The macula is a small but vital area located in the central portion of the retina, responsible for high-resolution, central vision [15]. In the center of the macular, lies the fovea, densely packed with cone photoreceptors and crucial for tasks such as reading and recognizing faces. Lipofuscin accumulation is particularly detrimental in the fovea, due to the high density of photoreceptors and the critical role of this region in detailed vision. Over time, the buildup of lipofuscin in the RPE leads to the deterioration of these photoreceptors, resulting in the characteristic central vision loss. Surrounding the fovea is the parafovea, which, while having a slightly lower concentration of cones, still significantly contributes to central vision. The perifovea encircles the parafovea and marks the outer boundary of the macula with an even lower concentration of cones. Beyond the perifovea extends the rest of the retina, primarily composed of rod photoreceptors, which are more sensitive to light and crucial for peripheral and night vision (Fig. 2). This distinct organization of the macula, especially the fovea, enables it to process detailed visual information, distinguishing it from the retinal periphery which is more adapted for low-light and peripheral vision detection.

Age of onset is a marker: STGD tends to be more severe when it emerges at a younger age. Typically, the onset occurs during childhood or early adolescence, around 10 to 15 years of age [16]. Noble and Carr utilized the fundus appearance during the initial presentation to categorize the disease into four distinct types: (1) macular degeneration without flecks; (2) macular degeneration with perifoveal flecks; (3) macular degeneration with widespread flecks; and (4) widespread flecks without macular degeneration [17]. Patients’ peripheral vision remains largely unaffected, so they can maintain their ability to navigate. Patients with STGD typically retain their peripheral vision, which allows them to navigate spaces and environments. However, the significant loss of central vision, a key disability in severe cases of STGD, often hinders their ability to perform tasks that require detailed, focused vision. This impairment can be particularly challenging in many work settings, where reading, computer use, and visual precision are essential. Consequently, individuals with severe STGD may find themselves unable to continue in their current jobs or careers, especially in the absence of sufficient workplace accommodations to mitigate their visual limitations. This often leads to reduced income, limited career advancement opportunities, and a compromised financial future, emphasizing the need for greater support and resources for individuals affected by such visual impairments in the workforce. The resultant loss of money is not confined to sheer dollars and cents; it extends to the loss of aspirations and dreams, which highlights the need for further research and therapeutic interventions to restore hope to those affected by this devastating condition.

### The genetic landscape

The disease is inherited in an autosomal recessive pattern and is linked to sequence variations in the *ABCA4* gene, which contains at least 50 exons and spans ~ 150 kb [18]. It is a large target for the accumulation of mutations, and the carrier frequency for these variations is estimated to be as high as 1 in 20 [19]. Notably, disease-causing variants are also correlated with conditions such as cone dystrophy, cone-rod dystrophy, and rod-cone dystrophy. *ABCA4*, a huge and highly polymorphic gene made up of 50 exons, has more than 900 disease-associated variants documented thus far [20]. It belongs to the extensive ABC transporter superfamily and is responsible for encoding the ABCA4 protein, which is a part of the ATP-binding cassette transporter superfamily [8]. *ABCA4*, predominantly located at the periphery of rod and cone outer segment discs, is instrumental in the process of protein synthesis and accumulation, where it actively transports retinoids from the photoreceptors to the RPE [21].

The disease exhibits considerable genetic heterogeneity, meaning that a wide range of mutations in the *ABCA4* gene can lead to STGD. This contributes to variations in the disease’s onset, severity, and progression among individuals. One notable example of this heterogeneity is the G1961E mutation in the *ABCA4* gene. This specific mutation is the most common among STGD patients, accounting for approximately 15% of all cases [22]. However, the penetrance of STGD is not always 100%, meaning that some individuals who carry disease-associated alleles may not develop clinical symptoms or may have very mild or late-onset manifestations [16]. The age at which STGD manifests and the severity of vision loss varies among affected individuals. This variability is influenced by a combination of genetic, environmental, and individual factors [23]. Further, the age at which an individual receives a diagnosis and how STGD is managed upon diagnosis is critical. Early diagnosis and proactive care can help to preserve vision and delay the severe loss of vision. Stressors on the retina, such as oxidative stress, can accelerate the degeneration process, and the presence of other ocular or systemic conditions may also influence the rate of vision decline.

The diagnosis of STGD is primarily reliant on clinical observations, particularly the distinctive changes observed in the ocular fundus. However, the role of electrophysiological evaluations in diagnosing STGD is not just confirmatory; these tests provide critical insights into the extent of retinal dysfunction and, consequently, valuable prognostic information [24]. The methodologies used, including multifocal, pattern, and full-field electroretinography (ERG), offer a better understanding of the disease’s impact on retinal activity. A classification system developed by Lois and colleagues (2001) is pivotal in categorizing STGD into three distinct phenotypic subtypes, each reflecting a different level of retinal impairment and potential disease progression. In Group 1, a severe pattern ERG abnormality indicative of macular dysfunction coexists with normal full-field ERGs. This suggests that, initially, STGD may predominantly affect central vision, sparing peripheral visual function. In contrast, Group 2’s generalized loss of cone function signifies a broader impairment, extending beyond the macula to affect color vision and VA more extensively. This progression reflects a more severe disease impact, likely translating to greater challenges in tasks requiring detailed and color vision. Group 3, marked by a generalized loss of both cone and rod function, indicates the most advanced stage of STGD, where both central and peripheral vision are compromised. This stage represents a significant escalation in the disease’s severity, potentially leading to a significant loss of visual function.

### Precision medicine

Understanding these distinct phenotypic presentations is crucial for tailoring patient prognosis and management. For Group 1, interventions focus on preserving macular health, with a focus on gene therapy for specific mutations and pharmacological treatments to protect the macula. In contrast, for Groups 2 and 3, since there is a generalized loss of cone and rod function, the strategies extend to broader retinal protection and repair, including stem cell therapy and comprehensive visual rehabilitation to adapt to more significant vision loss. As the treatment strategies for Groups 2 and 3 evolve to encompass broader retinal protection and more comprehensive approaches to adapt to extensive vision loss, the challenge of a lack of an established cure or universally effective treatment remains.

While research into potential therapies continues to advance, the complexity of STGD, with its intricate genetics and varying phenotypes, poses a barrier. However, ongoing studies are exploring promising approaches, including gene replacement therapy, stem cell therapy, and drug therapy, offering hope for effective treatments in the future. In the meantime, an emphasis on early detection and disease management becomes increasingly critical to slow disease progression and preserve remaining vision. Collaborative efforts among researchers, clinicians, and patients are essential to driving progress and finding solutions that can ultimately transform the lives of those affected by this disease.

Over the past few years, precision medicine has been seen as a promising approach to addressing this disorder. While there are drugs available that slow the progression of other types of macular degeneration, such as AMD, their effectiveness in STGD is limited. This difference in efficacy stems from the distinct pathophysiological mechanisms of STGD compared to other retinopathies [25]. In STGD, the primary issue is the accumulation of lipofuscin in the RPE due to *ABCA4* gene mutations, a process quite different from the predominantly age-related changes seen in AMD. Therefore, treatments effective in AMD may not address the genetic and cellular dysfunctions at the heart of STGD. This distinct genetic basis of STGD makes it more intractable compared to other forms of retinopathy where the disease mechanism may be more responsive to existing pharmacological interventions. Thus, the development of treatments for STGD requires a more targeted approach, focusing on the specific genetic and molecular abnormalities involved.

### Current treatments and lifestyle adaptations

As of 2023, the available treatments for STGD largely center around managing symptoms and adapting lifestyles without addressing the underlying genetic cause. Patients typically use low vision aids and make lifestyle modifications, such as UV light protection and a healthy diet, to mitigate the impact of vision loss. Some may also use vitamin supplements to support overall eye health. However, these approaches are fundamentally limited: they are non-curative and do not halt or reverse the disease’s progression. Their effectiveness is restricted to improving quality of life rather than preventing vision deterioration. Further, these treatments are not personalized to individual genetic profiles, an essential aspect considering the genetic variability in STGD. The landscape of treatment for STGD is poised for dramatic changes in the next 5–10 years. Advances in research, particularly in gene therapy and stem cell treatment, show promise in targeting the genetic underpinnings of the disease more directly. The evolution of precision medicine offers hope for more personalized and effective treatment strategies, potentially improving outcomes for STGD patients.

These therapies successfully corrected the underlying genetic defects and restored vision in animal models. However, proof in humans is needed. These innovative treatments offer exciting possibilities, as gene therapy seeks to correct the underlying genetic mutations responsible for *ABCA4* retinopathy by halting or even reversing the disease’s progression. Drug therapies aim to slow down the degeneration of photoreceptor cells and the accumulation of lipofuscin. Meanwhile, stem cell therapy has the potential to regenerate damaged retinal tissue, restore lost vision, and ultimately revolutionize the treatment landscape. Herein, I provide a review of the latest advancements in precision medicine for *ABCA4* retinopathy, specifically on the applications of gene, drug, and stem cell therapies, offering insights into the path forward as we seek to transform the lives of those living with this retinal disorder.

## Methods

A systematic literature review was conducted utilizing PubMed as the primary database. The review honed in on articles originally published in English from a range of nations, including the United States, United Kingdom, Germany, Australia, Japan, South Korea, and China. These countries were strategically chosen due to their active research communities dedicated to IRDs, notably STGD. In order to focus on the latest and most innovative therapeutic approaches, literature pertaining to these approaches was systematically limited to works with a publication date within the last ten years. An overview of the systematic literature review process, including the focused search categories and the specific keywords used was compiled into a table (Table 1). Details from each clinical trial were retrieved from ClinicalTrials.gov (US).

**Table 1:** Comprehensive literature search strategy for STGD therapies and diagnostics

Category	Search Focus	Keywords and Topics	Number of Citations
Clinical Interventions and Therapies	Drug therapies	- Drug therapies STGD	98 (17)
	Surgical interventions	- Surgical interventions STGD	32 (6)
	*ABCA4* gene therapy	- *ABCA4* gene therapy	126 (15)
	Retinal gene replacement	- Retinal gene replacement	880 (11)
	Stem cell therapy for Stargardt disease	- Stem cell therapy STGD	65 (32)
	Pharmacological interventions for macular degeneration	- Pharmacological interventions for macular degeneration	1,317 (5)
Genetic and Molecular Research	Underlying genetic mutations	- Underlying genetic mutations STGD	44 (8)
	Molecular pathways	- Molecular pathways STGD	24 (3)
	Gene therapies	- Gene therapies STGD	137 (14)
	Molecular mechanisms of retinal degeneration	- Molecular mechanisms of retinal degeneration	1,914 (7)
	Molecular pathogenesis of Stargardt disease	- Molecular pathogenesis of STGD	243 (19)
	Genetic variants in ABCA4	- Genetic variants in *ABCA4*	331 (21)
Early STGD Diagnosis	Early diagnosis	- Early STGD diagnosis	134 (9)
		- Early onset macular degeneration	518 (6)
		- Ophthalmological biomarkers for early diagnosis STGD	3 (2)
		- Early stage vision loss diagnosis	710 (17)
		- STGD screening	428 (4)
		- Vision loss in childhood diagnosis	713 (3)

The systematic approach to the literature review, outlining the different search categories, their focuses, and the specific keywords and topics used to gather relevant information on clinical interventions, genetic research, and early diagnosis of STGD. Keywords and topics within each category guided the retrieval of relevant articles from PubMed NCBI, with the number of citations reflecting the volume of research available for each search term. The number in parenthesis is the number of papers read

After these preliminary searches, the list of articles was further narrowed by only including publications that emphasized one of the following criteria: therapeutic innovation, clinical trial relevance, clinical applicability, *ABCA4-*associated retinopathies, and/or human genome. Given the extensive body of literature in this field, it was challenging to conduct a thorough review of the literature. The chosen works for this review are deemed representative of the latest advancements in precision medicine therapies for STGD.

### Drug therapies for STGD

Drug therapies aim to address the underlying cellular dysfunction, such as the accumulation of toxic substances like lipofuscin in the retina or the degeneration of photoreceptor cells. These types of therapies are advantageous because they have the potential to stabilize or slow the progression of the disease through systemic treatment. Researchers are investigating various compounds that can reduce visual cell damage, manage oxidative stress, or improve the metabolic health of retinal cells. Currently, there are numerous clinical trials that explore compound therapies that target the critical pathological processes associated with STGD [26]. As of the current “state-of-the-science,” there are no drug therapies specifically approved for STGD. Treatment mainly focuses on managing symptoms and preventing further deterioration. Patients are advised to avoid high-light environments and use protective lenses to shield their eyes from potential damage. Behavioral modifications, such as using low vision aids and making lifestyle adjustments, are recommended in order to enhance daily functioning and preserve the remaining vision. Research continues into pharmacological treatments, but as of now, the focus remains on supportive care and mitigating the impact of the disease on patients’ lives.

The link between lipofuscin accumulation and complement system activation in RPE cells, a layer of cells behind the retina that nourish photoreceptor cells, as observed in the *ABCA4* knockout model, extends beyond cell lines to more complex organisms [27]. Since complement activation plays a significant role in the development of AMD, the similar characteristics shared between this condition and STGD suggest that medications developed for AMD could also be applicable to STGD [28]. The translational research, bridging the gap from cellular studies to more advanced models, often employs animal models such as mice genetically modified to mimic STGD, and in some cases, primate or porcine models, to replicate the disease’s progression and test potential treatments. Avacincaptad pegol (Zimura), a complement inhibitor, exemplifies this translational journey. Initially tested in cell lines and animal models, Zimura’s effectiveness in inhibiting the C5 component of the complement system and its downstream effects, C5a and C5b, has been established. Its intravitreous injection reduces chronic inflammation and cell death in the macula, thereby slowing the degeneration of RPE cells [29]. This pathway from basic research to clinical application demonstrates the interconnectedness of various research methodologies in developing treatments for complex diseases like STGD.

An ongoing Phase 2b clinical trial is currently underway to evaluate the safety and effectiveness of a Zimura intravitreal injection compared to a Sham (placebo-like) injection in patients with STGD. Patients of all sexes aged 18–60 are eligible for this study, and their best-corrected visual acuity (BCVA) must be between 20/20 and 20/200 (National Library of Medicine [NLM], NCT03364153). Approximately 120 people from around the world are enrolled in the study in 35 centers, and each participant will receive 36 injections. The study will continue for approximately 18 months; results have not yet been reported. A Phase II/III trial in 286 patients with AMD found that Zimura was well-tolerated and effective (NLM, NCT02686658). The participants will be randomly assigned to one of the following groups: Zimura and Sham (an empty syringe with no needle to prevent bias in results).

Vitamin A (all-*trans*-retinol) is a common target of the visual cycle and is a precursor to 11-*cis*-retinol. It is presumed that decreasing vitamin A levels may inhibit the synthesis of all-*trans*-retinol and, consequently, lipofuscin [26]. Although conventional vitamin A supplements could be harmful, as seen in the *ABCA4* knockout model involving genetically modified mice where the *ABCA4* gene is inactivated [27], an alternative strategy involves administering deuterated vitamin A (ALK-001). ALK-001 (gildeuretinol) is an altered form of vitamin A that slowly forms vitamin A dimers in the body by replacing its natural form [30]. It is the first medicine that is being clinically developed to treat STGD. Compared to traditional vitamin A supplementation, ALK-001 can minimize the formation of toxic vitamin A dimers that contribute to retinal degradation in STGD. This occurs by modifying the structure of the vitamin A molecule so that three hydrogen atoms are replaced with deuterium atoms. ALK-001’s modified structure allows it to specifically target and deliver vitamin A to the retina [30]. Researchers have found that ALK-001 slowed the rate of dimerization of vitamin A by 4–5 times in in vitro studies and by 80% in in vivo mouse models, which leads to the preservation of visual function in animal models of STGD [31]. An active clinical trial is currently in Phase 2 to evaluate how orally administered ALK-001 affects the progression of *ABCA4*-related STGD. Participants in the study can be male or female and must be between 8 and 70 years old with any VA. According to the results, the growth rate of atrophic lesions in the ALK-001-treated group was 21% slower than that in the untreated group (NLM, NCT02402660). Furthermore, the results of the study have shown that this is the first instance where a therapeutic intervention has demonstrated a clinically and statistically significant slowdown in the progression of STGD.

Building on the theme of targeting vitamin A-related pathways in STGD, other therapeutic approaches are being explored. Tinlarebant (LBS-008) is one such oral drug therapy. It operates by inhibiting the activity of RBP4 (retinol-binding protein 4), which is responsible for transporting retinol to the eye [32]. By reducing the transport of retinol, Tinlarebant aims to decrease the accumulation of harmful substances in the retina, thereby lowering the risk of blindness associated with STGD. An ongoing clinical trial currently in Phase 3 is assessing how effective Tinlarebant is in slowing the progression of atrophic lesions in adolescents with STGD. Participants will take 5 mg of Tinlarebant/placebo for up to 24 months (NLM, NCT05244304).

Additionally, metformin hydrochloride, commonly prescribed for the treatment of type 2 diabetes, is being investigated for its potential benefits in retinal degeneration. A correlation between metformin use and the slowing of the progression of retinal degeneration has been discovered because metformin increases macroautophagy through the mammalian target of the rapamycin complex 1/AMP-activated kinase pathway [33]. The stimulation of this pathway allows the RPE to better handle lipofuscin. This approach is being tested in a Phase 2 clinical trial to determine its safety and efficacy in slowing the rate of change in the degeneration of photoreceptors in *ABCA4* retinopathy (NLM, NCT04545736).

The realm of drug therapies for STGD is witnessing a significant evolution, with several promising strategies currently being investigated. From the use of modified vitamin A derivatives such as ALK-001 to innovative approaches such as Tinlarebant that inhibits the transport of retinol to the eye and the repurposing of metformin to slow retinal degeneration, the landscape of treatment options is expanding. These therapies, each with their unique mechanisms of action, are at various stages of clinical trials and offer hope for more effective management of STGD. The results of these studies could not only revolutionize the treatment of this specific retinal disease but also provide valuable insights into managing other similar retinal disorders, paving the way for a future where vision loss due to genetic conditions can be significantly mitigated or even prevented.

### Gene therapies for STGD

Since a significant portion (90–95%) of STGD cases stem from loss-of-function mutations in the *ABCA4* gene and are inherited in an autosomal recessive manner, gene therapy presents a logical strategy to both prevent and slow the progression of retinopathy associated with *ABCA4* [34]. Every year, there are new ongoing clinical trials for gene therapy treatments for IRDs in which a pathogenic gene is replaced with a functional gene [35]. Furthermore, delivering large DNA molecules, which is required for treating Stargardt disease, remains a challenge. This is due to the preferred vector being adeno-associated virus (AAV), a small, single-stranded DNA dependovirus. This virus belongs to the parvovirus family and has a packaging capacity of ~ 4.7 kb [36]. Therefore, strategies to facilitate large gene delivery, such as dual-AAV, lentiviral, and nonviral platforms, are being developed.

### Lentiviral vectors

Since the human *ABCA4* cDNA is almost 7 kb, lentiviral vectors are an appropriate choice for gene therapy because they have a packaging capacity of ~ 8 kb and carry large expression cassettes [37]. They deliver therapeutic genes into the host genome of transduced cells in vivo and transduce nondividing cells, which is necessary for terminally differentiated cells such as photoreceptors [38]. These vectors have the potential to halt or reverse the accumulation of lipofuscin, preserving photoreceptor function over time. The targeted delivery of lentiviral vectors to the retina can be achieved through subretinal injections, a method that has been refined to increase precision and reduce invasiveness. Furthermore, the development of lentiviral vectors that express *ABCA4* under the control of retina-specific promoters can enhance the specificity of expression, reducing the possibility of off-target effects and maximizing therapeutic benefits.

In the field of eye diseases, the unique aspect of using one eye as a control in clinical trials is particularly intriguing. This approach was evident in the clinical application of a novel gene therapy technology, where a subretinal injection of EIAV-*ABCA4* (equine infectious anemia virus) was administered to the worse-seeing eye of 22 patients. They were followed for three years to assess the safety and efficacy of this treatment. The use of the worse-seeing eye as the treatment site allows for direct comparison with the patient’s other eye, serving as a natural control. SAR422459, the drug used in this study sponsored by Sanofi (NLM, NCT01367444), exemplifies the lentiviral vector’s potential. It utilizes a self-inactivating EIAV vector designed to deliver the *ABCA4* gene directly to the affected retinal cells. While 27% of treated eyes experienced worsening of retinal pigment epithelium atrophy as observed on fundus autofluorescence, an imaging technique that displays retinal changes, there was also a noteworthy positive outcome. One treated eye from the cohort receiving the highest dose experienced a reduction in macular flecks. This study was discontinued following a reassessment of clinical development objectives. However, the initial safety outcomes observed over the first year were encouraging. Participants reported 125 adverse events, all of which were classified as mild to moderate in severity [39]. A second long-term dose-escalation phase I/II trial explores subretinal injection of an *ABCA4*-carrying lentivirus in 27 patients (NLM, NCT01736592).

### Adeno-associated viral vectors

In parallel, the use of adeno-associated viral (AAV) vectors has also gained prominence in gene therapy research for retinal diseases. Over the past two decades, AAV vectors have been a focal point due to their safety profile and efficiency in gene delivery [40]. These vectors are being extensively studied for a variety of IRDSs, including STGD, offering another avenue for therapeutic intervention. The exploration of different viral vectors - lentiviral and AAV - reflects the dynamic nature of gene therapy research in ophthalmology. Each vector type has unique characteristics and potential applications, contributing to the broader goal of developing effective gene therapies.

AAVs have emerged as the preferred vector for gene therapy due to their favorable safety profile with few adverse effects [35]. However, a significant hurdle persists: the limited capacity of AAV vectors to carry only small genes, which leaves many monogenic diseases untreatable using existing technologies. Initial techniques focused on engineering a single large transgene with the complete 6.8 kb sequence of the *ABCA4* gene, and the first AAV vector-based gene therapy product for an IRD was approved in December 2017. However, the addition of necessary regulatory sequences increased the size to over 10 kb, beyond the typical packaging limit of AAV vectors. Despite this, researchers managed to encapsulate and express the full-length ABCA4 protein in vitro and in vivo [41]. The AAV packaging process, however, is unregulated and results in varied incomplete transgene versions within the vector shells, known as a fragmented dual-vector approach [42]. The effectiveness of this method depended on serendipitous overlaps between the truncated transgenes [43]. Further assessments showed that the fragmented AAV-ABCA4 vectors could recombine to create the full *ABCA4* gene transcript but generated hybrid transcripts with unintentional AAV genome integrations [44]. Although initial attempts to package an oversized AAV transgene with the complete *ABCA4* were successful, it became clear that this strategy was not reasonable because it is challenging to produce homogeneous AAV preparations containing the intended transgene. Hence, the focus shifted to dual AAV vector strategies in light of the potential therapeutic advantages of delivering a functional *ABCA4* gene.

The advancements in using AAV vectors for gene therapy, particularly for conditions like STGD, have originated from a combination of laboratory research using human cell lines and in vivo studies in animal models. In labs, researchers often use human retinal cell lines, including RPE cells, to study the behavior of AAV vectors and the expression of the *ABCA4* gene. These cell lines are important for understanding how the therapy might work in human tissues and for testing the safety and efficacy of different vector designs. Mouse models play a significant role in gene therapy research. Mice genetically modified to mimic the genetic and physiological aspects of STGD provide a platform for testing AAV vectors in a living organism. These models allow researchers to observe how the therapy affects the retina over time and to identify potential side effects or complications. Initial strategies focused on engineering a single large transgene with the complete sequence of the *ABCA4* gene. The challenge of including necessary regulatory sequences and the limitations of AAV vectors’ capacity led to the development of fragmented and dual-vector approaches.

Dual AAV vectors are a method used in gene therapy when the gene fixed into a patient’s cells is too big for one virus to carry. If the gene cannot fit into a single AAV vector, scientists split it into halves (< 5 kb in size) [45]. One vector contains the promoter and the 5’ portion of the cDNA, while the other contains the polyA signal and the 3’ portion [46]. Once inside the target retinal cells, these split gene segments can reassemble into the complete, functioning gene. This reassembled *ABCA4* gene can produce the correct protein, which is vital for the visual cycle and prevents the accumulation of lipofuscin and bisretinoid. The results of a study in mouse models demonstrate that delivering the ABCA4 protein via dual AAV vectors significantly reduces lipofuscin in the *ABCA4* animal model of STGD [46].

AAVantgarde Bio, an Italian headquartered biotechnology company, is working to validate an AAV-based large gene delivery platform named AAV intein. Inteins carry out a process known as protein splicing, a multi-step biochemical reaction made up of the cleavage and formation of peptide bonds [47]. AAV intein vectors mediate the expression of large therapeutic proteins in the retina and in human cell lines derived from RPE cells. In STGD mouse models, subretinal administration of AAV intein vectors improved the retinal phenotype, which demonstrated the potential of AAV intein for gene therapy of these types of blinding diseases [48]. Preclinical studies are currently ongoing for the AAV intein platform. Preliminary results presented at the 2024 Association for Research in Vision and Ophthalmology (ARVO) showed positive non-human primate (NHP) safety and efficacy data at demonstrating that the intein vectors can be administered safely to NHPs. This confirms that the AAV intein-mediated retinal gene therapy for STGD is safe and effective in relevant animal models, moving closer towards the clinical translation of this platform.

Similarly, other researchers are developing innovative strategies to overcome the limitations of using a single AAV vector. The American biotechnology company Applied Genetics Technologies Corporation (AGTC) also announced a dual vector gene therapy system in 2019 developed by researchers from the Department of Ophthalmology at the University of Florida designed to accommodate larger genes within the retina, such as *ABCA4*. The two DNA fragments recombine once inside the cell and reconstruct the entirety of the *ABCA4* coding sequence. The researchers found that after subretinal injection of dual AAV into KO mice lacking the *ABCA4* gene, the full-length ABCA4 protein correctly localized to photoreceptor outer segments. These outer segments are key structures in photoreceptor cells because they convert light into visual signals and are the primary site where the ABCA4 protein functions in the visual process [7]. Furthermore, there was a reduction in the accumulation of lipofuscin. ATGC is moving forward with the preclinical development of the therapy, having shared data that demonstrates that it is effective in non-human primates [46]. William Hauswirth, the leader of the research team at UF, has pointed out the significance of AGTC’s success in safely delivering and expressing the ABCA4 protein in these primates through subretinal injection using their optimized vectors.

**Fig. 3:**
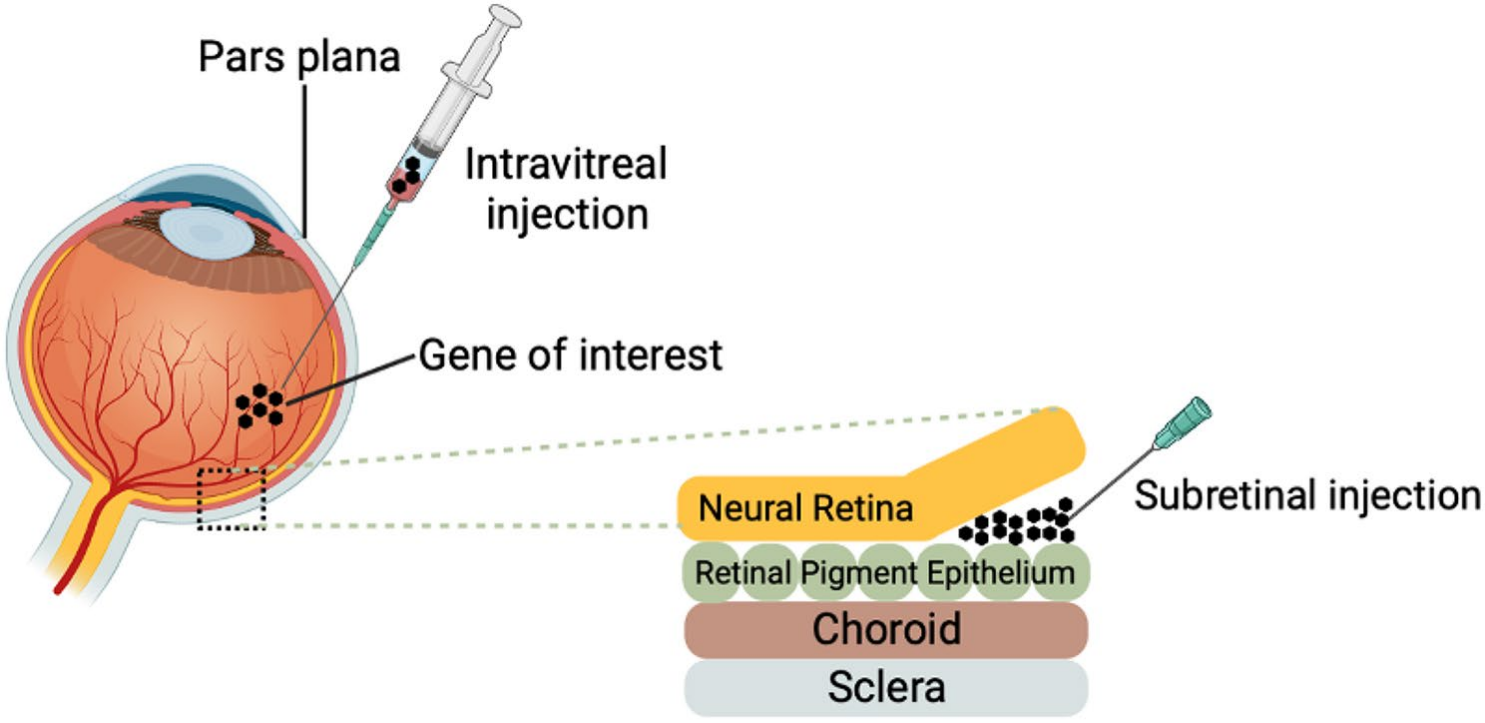
Intravitreal and subretinal injections are currently the most used techniques for viral vector delivery.

In a similar pursuit of innovation, ViGeneron has three novel next-generation gene therapy platforms that aim to target the drawbacks of current AAV therapies: vgAAV vector platform, REVeRT technology platform, and AAV Transactivation. The vgAAV vector platform enhances the transduction efficiency of target cells, facilitating minimally invasive administration methods such as intravitreal injections (Fig. 3). The second platform is REVeRT (REconstitution Via mRNA Trans-splicing) technology, which enables large genes (> 5 kb) to be effectively reconstituted in tissues, including the heart, brain, liver, skeletal muscle, and retina. In a mouse model of STGD, the researchers demonstrated high expression of *ABCA4*. Preliminary experimental data has suggested a potential improvement in retinal function. Finally, AAV transactivation utilizes CRISPR‒Cas technology to allow for the in vivo regulation of one or more genes. Its program, VG801, focuses on transferring the *ABCA4* gene in a mouse model for STGD. The gene was transferred using REVeRT technology to account for the large size of *ABCA4*.

### Nonviral vectors

Additionally, despite the success of viral gene delivery in clinical trials, issues related to host immune responses and oncogenesis have been reported. As the industry continues to explore the full potential of AAV vectors, attention is also shifting toward the promising realm of nonviral delivery systems. These systems are particularly compelling due to their ability to overcome some of the inherent limitations of viral vectors, such as the restriction on the size of the genetic payload and concerns about immune responses. Intergalactic Therapeutics uses synthetic biology and engineered gene circuits to deliver vectors in a nonviral way by making covalently closed and circular DNA molecules (C3DNA). C3DNA enables DNA vectors that are too large for viral delivery systems to be delivered without being inserted into the host genome, thus reducing the risk of insertional mutagenesis [49]. The data from its lead program, IG-002, demonstrated that subretinal administration of a DNA payload that encodes the human *ABCA4* gene expressed human ABCA4 protein in adult porcine retinas over the course of 12 months. The C3DNA system, in combination with COMET delivery, shows great potential for gene therapy, especially for diseases requiring large DNA payloads. Intergalactic has plans to move its program into the clinic in 2024. This advancement could broaden the scope of treatable genetic conditions, signaling a new chapter in gene therapy that complements the ongoing successes of viral vector-based approaches such as those pursued by Ocugen.

### Base editing

**Fig. 4:**
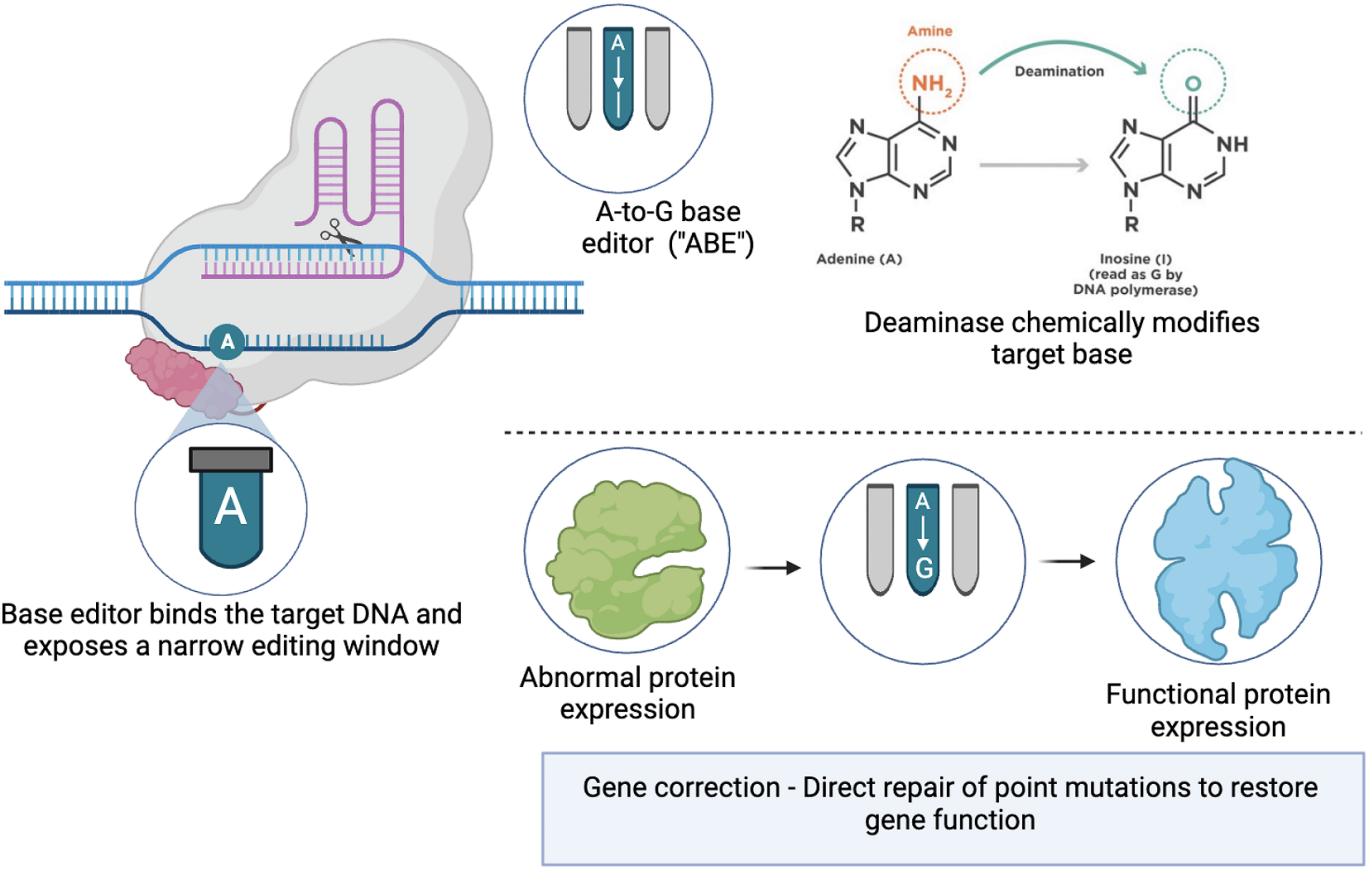
This figure illustrates the process by which Beam Therapeutics’ base editors achieve precise and permanent single nucleotide substitutions within the genome. The base editor, depicted as a complex binding to the target DNA strand, exposes a narrow editing window where a specific adenine (A) base is targeted. A deaminase enzyme is then utilized to chemically modify the adenine into inosine (I), which is read as guanine (G) by the cell’s DNA polymerase, converting an A-T base pair into a G-C base pair. This process corrects point mutations in the DNA sequence, leading to the expression of a functional protein instead of an abnormal one and restoring gene function without the need for double-stranded DNA breaks.

The evolving landscape of gene therapy paves the way for advanced genome editing techniques such as base editing. Base editing is different from other gene editing tools, such as CRISPR-Cas systems that typically create double-stranded breaks in DNA. Base editing offers a more targeted approach than traditional gene therapies, allowing for the direct, irreversible conversion of one DNA base into another, effectively correcting point mutations without making double-stranded DNA breaks (Fig. 4) [50]. This process is similar to fixing a single typo in a text. CRISPR-Cas-based editing systems have also been adapted for base editing purposes. These adaptations involve coupling the CRISPR machinery with enzymes that can modify DNA bases directly, thus enabling precise alterations at the genetic level without the need for breaking and repairing the DNA strands [51].

The Institute of Molecular and Clinical Ophthalmology Basel is working with Beam Therapeutics in Boston, Massachusetts to bring this innovative technology to the clinic. The goal of their project is to transfer the specific base-editor complex to patients’ retinas in the hopes of correcting the *G1961E* mutation. Base editing works by introducing single-nucleotide variants into DNA or RNA in living cells [52]. Mutations in the *G1961E* gene lead to the death of photoreceptors and RPE, and the demonstration of precise base correction in a high percentage of target cells is an essential requirement for the therapeutic application of base editing. Adenine base editing is a rational therapy for correcting the most common mutation in STGD because, at the optimal dose level, the optimized gene therapy vector in the study led to 40% editing rates in cones, 28% in rods, and 73% in RPE cells [53].

Overall, base editing is a highly promising development in the field of gene therapy, but whether it is more promising than the therapies previously mentioned in this review depends on various factors, including the mutation type and the maturity of the technology. Even though there are reports demonstrating the effectiveness of base editing in cell lines and mice, high levels of gene correction have not yet been demonstrated in human tissues and non-human primates [53]. Furthermore, the applicability of base editing is currently limited to certain types of mutations, such as transition mutations. Other therapies being developed, such as those utilizing AAV vectors or nonviral delivery methods like C3DNA, may be more suitable for a broader range of genetic alterations. However, whether it is more promising than the other gene therapies in this review cannot be fully determined until more data from comparative clinical trials becomes available.

### Optogenetics

**Fig. 5:**
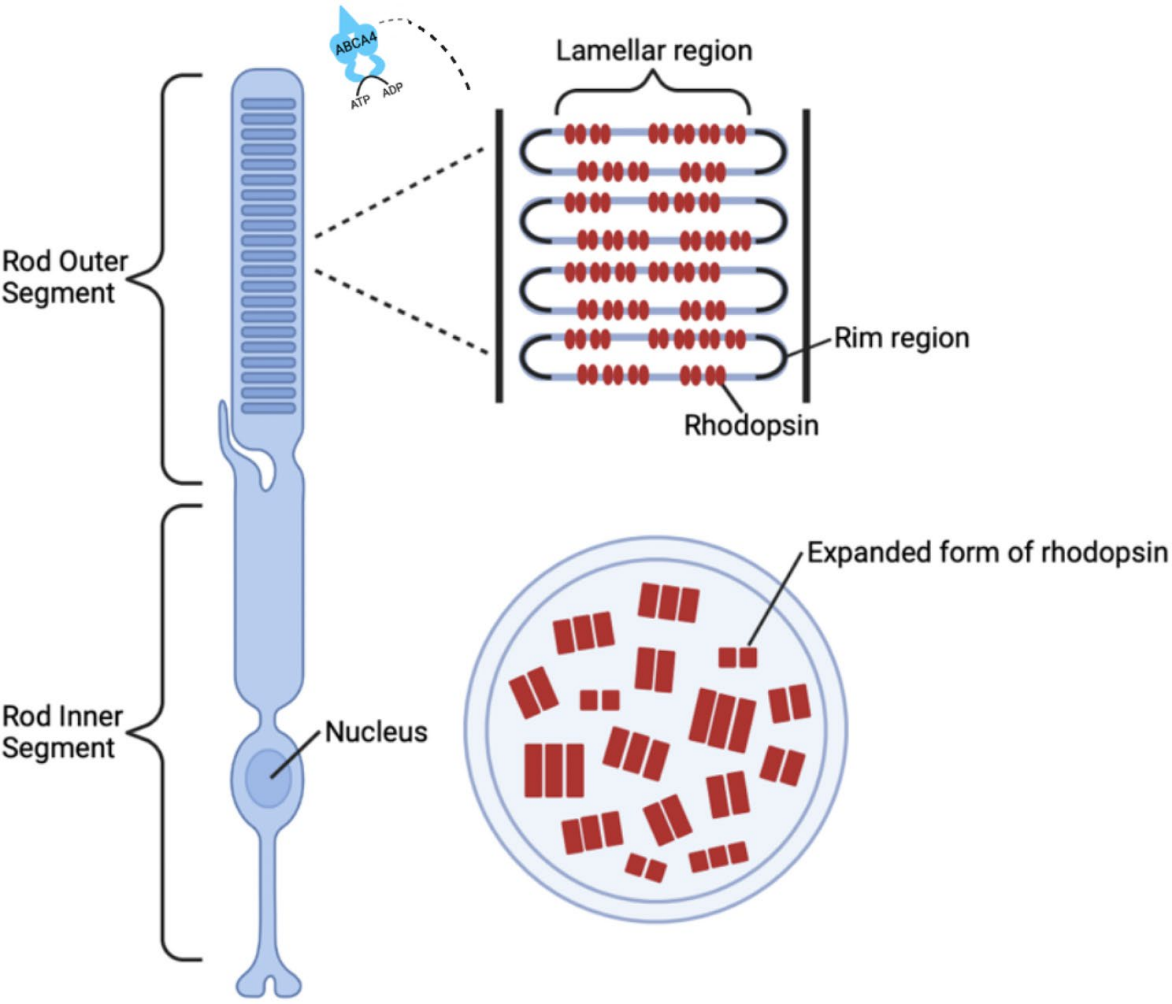
This diagram visualizes the structural components of rod photoreceptor cells in the retina, emphasizing the significance of the location of the *ABCA4* gene in relation to STGD gene therapies. The *ABCA4* protein, active in the rod outer segment, is essential for transporting retinoids as part of the visual cycle. Mutations in this gene disrupt this process, leading to the accumulation of toxic substances and contributing to STGD pathology. Rhodopsin, the light-sensitive protein within the lamellar and rim regions, is depicted in its normal and expanded forms to illustrate the structural changes that mutations can cause.

Optogenetics is a groundbreaking technique that merges genetic engineering with light to control the activity of individual neurons within living tissue [54]. This method involves the introduction of light-sensitive proteins, known as opsins, which are naturally occurring in certain algae and microorganisms, into specific types of cells, such as neurons in the retina [55]. Opsins are membrane proteins found in the photoreceptor cells of the retina (Fig. 5). Light interacts with opsins when it enters the eye and reaches the retina, which generates electrical signals that are transmitted to the brain by the optic nerve to be interpreted as visual information [56]. Nanoscope Therapeutics Inc. has come out with a novel therapeutic, Multi-Characteristic Opsin (vMCO-010), an ambient light activatable optogenetic therapy that works to restore vision in the advanced stages of STGD by restoring broadband light sensitivity, the ability of these introduced opsins to respond to a wide range of light wavelengths [56]. This broad sensitivity allows treated retinal cells, which are not primarily light-sensitive, to capture a wider spectrum of light, mimicking the function of healthy photoreceptors and generating neural signals for visual perception. A Phase 2 clinical trial completed in September 2023 evaluated the safety and efficacy of a single intravitreal injection of MCO-010 in 6 subjects with STGD (NLM, NCT05417126). The results were promising; patients demonstrated clinically meaningful improvements in BCVA, and there was a ~ 3 dB gain in mean light sensitivity measured by Octopus visual field perimetry.

### Cell replacement therapy for STGD

As the degeneration of RPE and photoreceptor cells leads to diminished VA in the advanced stages of STGD, the treatment approaches discussed so far primarily aim to halt further vision loss. In order to restore vision that has already been lost, transplanting new cells is necessary. Encouraging proof-of-principle data has emerged from pre-clinical studies that involve introducing human embryonic stem cell (hESC)-derived RPE cells into the subretinal space of mice [57]. Regenerative medicine holds immense potential benefits for patients suffering from blinding eye diseases. Due to their unlimited proliferation and ability to differentiate into various cell types, stem cells are a valuable resource for tissue transplantation, and this type of therapy has the potential to treat STGD by regenerating the RPE. The stem cells are classified based on their potency, which refers to the range of cell types they can differentiate into and their origin [58]. The use of stem cells in therapy encompasses various approaches, with cell replacement therapy being the most recognized. In this method, stem cells are transformed into the specific cell type needed and then transplanted into the damaged tissue, where they integrate and work to restore its function [59].

### Embryonic stem cells

Embryonic stem cells (ESCs) are a source of therapeutic cells that can treat diseases due to dysfunction or tissue loss. Using this technology to treat diseases that impact the eye is an appealing first-in-human application. Genetically altered photoreceptor outer segments are often the cause of RPE degeneration in STGD and in preclinical macular degeneration models, which often utilize genetically modified animal subjects or cell cultures to replicate aspects of the disease, there is proof that subretinal transplantation of hESC-derived retinal pigment epithelium can save photoreceptors and stop vision loss [60]. The transplanted hESC-derived RPE cells provided the necessary support and functionality that the damaged native RPE cells could no longer offer. As a result, this intervention was shown to preserve photoreceptor cells and halt or slow down the progression of vision loss in these models. This study provided essential insights into the possibility of using stem cell-derived RPE cells for treating retinal diseases. It laid the groundwork for future studies and clinical trials and offered a new way to restore vision in diseases characterized by RPE degeneration. Following the development of the first hESC line by James Thompson and colleagues (1998), the application of hESC lines and their derivatives in both non-therapeutic and therapeutic contexts has ushered in a new era of innovation in modern medicine.

hESC-derived cell therapies have undergone intense scrutiny regarding ethical, regulatory, and biological safety aspects for human applications. After the Geron Corporation halted its pioneering hESC-based therapy for spinal cord injury in 2011, the Astellas Institute of Regenerative Medicine took the lead. They initiated the first hESC-derived RPE clinical trials for retinal degenerative diseases such as STGD and Geographic Atrophy (GA) due to AMD. Beginning in April 2011 and concluding in 2017, three Phase 1/2 clinical trials tested the MA09-hRPE cell line in suspension form across major eye centers in the United States, United Kingdom, and South Korea. These trials (UK-SMD: NCT01469832; US-SMD: NCT01345006; US-AMD: NCT01344993) have offered preliminary insights into the potential therapeutic benefits and behavior of the MA09-hRPE cell line in severely damaged subretinal environments in advanced STGD cases. Many patients in these trials had clinically measurable visual improvements [61]. By replacing the RPE cells lost in diseased retinas, hRPE transplants ultimately aim to restore the anatomical and functional integrity of the blood-retinal barrier (BRB) [62]. This restoration is crucial as the BRB is responsible for regulating ion, protein, and water flux into and out of the retina [63].

Advancements in multimodal imaging and functional testing, including adaptive optics-based scanning laser ophthalmoscopy and microperimetry, will be crucial in establishing whether transplanted cells directly improve visual function in specific retinal areas. Should such a direct impact be confirmed, subretinal transplants of stem cell-derived RPE transplants could emerge as the first effective treatment for a significant unmet medical need, potentially benefiting a large patient population.

## Discussion

The purpose of this literature review was to examine the advancements in drug, gene, and stem cell therapies for STGD, a prevalent juvenile macular dystrophy primarily caused by mutations in the *ABCA4* gene. Presently, individuals newly diagnosed with STGD face limited options, focusing mostly on symptom management and lifestyle adjustments. They rely on aids such as magnifiers and protective lenses, with clinicians closely monitoring the progression without a way to halt or reverse it. For those with advanced STGD, the current approach remains largely supportive and focuses on maximizing residual vision and adapting to significant vision impairment. Given the diversity of potential approaches for treating the disease, it can be challenging to design clinical trials and select patients because phenotype and rates of disease progression greatly vary [64]. However, the landscape is set to transform dramatically in the next five years. This shift will see a transition from managing symptoms to implementing personalized, proactive treatment strategies. In advanced STGD cases, the future holds the promise of innovative therapies, such as stem cell treatments and CRISPR-based techniques, offering the potential for vision restoration and a marked improvement in quality of life. Clinically, this means a move beyond supportive care to regular interventions with these advanced therapies.

The exploration of gene therapies, particularly those addressing the *ABCA4* gene mutations, marks a significant advancement in the quest to target the genetic root of STGD. Despite challenges in delivering large gene sequences, innovations like dual-AAV vectors and the AAV intein platform by AAVantgarde Bio demonstrate a promising direction. The key takeaway from these developments is the potential to directly correct the underlying genetic anomalies, offering a more permanent solution than symptomatic treatments. However, the complexity of STGD, with its extensive genetic heterogeneity, calls for a more tailored approach, considering the specific mutations present in each patient.

On the other hand, drug therapies provide more of a practical approach and are already available to manage the disease’s progression. The advancements in understanding the molecular pathways of STGD, particularly the role of vitamin A derivatives and complement inhibitors, have led to the development of drugs such as ALK-001 and Tinlarebant. These interventions aim to slow the degenerative process and preserve existing visual function, offering patients a better quality of life. While these therapies may not reverse the damage already done, their role in slowing disease progression and mitigating symptoms is invaluable.

Stem cell therapy, particularly the use of hESC-derived RPE cells, introduces a revolutionary potential to regenerate lost or damaged retinal tissue. This approach, standing at the frontier of regenerative medicine, can potentially halt the disease and reverse its effects. The preliminary success of transplanting hESC-derived RPE cells in animal models fosters hope for restoring vision in individuals with advanced STGD. Yet, the challenge remains in ensuring the integration, survival, and functional compatibility of these cells in the human retina.

To conclude, the fight against STGD is promising, with gene, drug, and stem cell therapies each contributing to a comprehensive treatment model. Each therapy brings unique strengths: drug therapies provide symptom management, gene therapies offer specificity, and stem cell therapies offer the potential of regeneration. The optimal strategy may lie in a combination of these approaches, tailored to individual patient needs based on genetic makeup, disease stage, and response to treatment. The integration of these therapies, informed by ongoing research and clinical trials, is essential in developing effective, personalized treatments. As we advance, the hope is not just to manage STGD but to restore vision and improve the lives of those affected by this challenging condition. The future of STGD treatment is a mosaic of these diverse yet complementary strategies, marking a new era in the management of IRDs.

## Data Availability

Not applicable.
